# Conscientious Objection to Harmful Animal Use within Veterinary and Other Biomedical Education

**DOI:** 10.3390/ani4010016

**Published:** 2014-01-21

**Authors:** Andrew Knight

**Affiliations:** Ross University School of Veterinary Medicine, P.O. Box 334, Basseterre, St Kitts, West Indies; E-Mail: aknight@rossvet.edu.kn; Tel.: +1-869-465-4161 (ext. 1143); Fax: +1-869-466-5206

**Keywords:** veterinary education, veterinary curriculum, conscientious objection, humane teaching methods, 3Rs

## Abstract

**Simple Summary:**

Classes in which animals are harmed are controversial within veterinary and other life and health sciences courses. Increasingly, students object to the harmful use of animals, and request humane teaching alternatives. Such cases can raise important animal welfare, legal and administrative concerns for universities. Several have implemented formal policies to guide their responses, maximising the likelihood of optimal and consistent outcomes. This paper reviews the development of these conscientious objection policies within Australian veterinary schools, and examines their underlying legal foundations. It concludes with recommendations for other universities considering how to respond to such cases.

**Abstract:**

Laboratory classes in which animals are seriously harmed or killed, or which use cadavers or body parts from ethically debatable sources, are controversial within veterinary and other biomedical curricula. Along with the development of more humane teaching methods, this has increasingly led to objections to participation in harmful animal use. Such cases raise a host of issues of importance to universities, including those pertaining to curricular design and course accreditation, and compliance with applicable animal welfare and antidiscrimination legislation. Accordingly, after detailed investigation, some universities have implemented formal policies to guide faculty responses to such cases, and to ensure that decisions are consistent and defensible from legal and other policy perspectives. However, many other institutions have not yet done so, instead dealing with such cases on an *ad hoc* basis as they arise. Among other undesirable outcomes this can lead to insufficient student and faculty preparation, suboptimal and inconsistent responses, and greater likelihood of legal challenge. Accordingly, this paper provides pertinent information about the evolution of conscientious objection policies within Australian veterinary schools, and about the jurisprudential bases for conscientious objection within Australia and the USA. It concludes with recommendations for the development and implementation of policy within this arena.

## 1. Introduction

Within veterinary education many thousands of animals have been killed worldwide during attempts to demonstrate scientific principles within preclinical years, or to teach practical skills within the clinical components of courses. Animals have been killed and dissected to demonstrate anatomical principles. Living animals or organs taken from them have been subjected to invasive experiments in physiology, biochemistry, pharmacology and parasitology laboratories. In many countries the mainstay of veterinary surgical practical training has been the practice of surgical procedures on healthy animals, which have frequently been killed following the procedure. Additionally, students in veterinary, animal science or other courses have often been required to assist with husbandry procedures on farm animals, such as teeth clipping in piglets, and castration in several species. These are usually legally sanctioned procedures, but because they can cause substantial pain and are frequently conducted without analgesia, they cause serious concerns for many students. Similarly, students may be required to attend abattoirs, although their role is usually observational rather than participatory.

However, alternative teaching methodologies have continued to develop for many of these educational applications, and databases including thousands of them now exist (e.g., NORINA: http://oslovet.norecopa.no). Such methods include computer simulations, high quality videos, ethically-sourced cadavers obtained from animals that have died naturally or in accidents, or most commonly, been euthanized for medical reasons, anatomical specimens preserved in a variety of ways, models, mannequins and surgical simulators, non-invasive self-experimentation, and supervised clinical experiences [1,2,3,4,5,6]. Social attitudes toward more traditional teaching exercises in which animals are harmed have also evolved in recent years and decades, concurrent with changes in student demographics and backgrounds. These factors have led to increasing desires among both students and faculty for more humane teaching methods, which are not reliant on harmful animal use. 

Such desires can be particularly strong in certain students, such as those with certain ethical, religious or other viewpoints or sensitivities. Unlike faculty members who have usually been immersed in veterinary school environments for much longer periods, students asked to seriously harm or kill animals during their education may well be confronting such issues for the first time. Indeed, for many it will actually be the first time they’ve been directly required to harm or kill an animal, to further their educational or career goals. For some students this may profoundly conflict with their personal ethics, and indeed, with the reasons they’ve chosen to embark on a veterinary degree in the first place.

However, faculty opposition to student requests for humane teaching methods has been common, and has sometimes resulted in conflict. Universities that refuse to provide alternatives for students can find themselves legally liable, and in some cases damages have resulted. In 1995 University of Colorado medical student Safia Rubaii sued her university for US$95,000 after failing physiology, because she refused to perform a compulsory experiment which required her to give a lethal injection to an anaesthetised dog. She was forced to retake physiology at the Creighton University School of Medicine in Nebraska, where harmful animal use was not required. Dr. Rubaii successfully graduated from her university in the same year she sued it. When upholding her legal claims, the court also required the university to provide alternatives to future students who might request them, and these terminal dog laboratories have since been entirely replaced by humane alternatives [7,8].

Considerable adverse publicity has sometimes resulted from such cases, when curricular animal killing and academic sanctions applied to conscientiously objecting students are publicised through the mass media. Such cases have the potential to significantly impact the reputations of universities, because of negative public viewpoints about the harming and killing of healthy animals within curricula, and also, because of negative viewpoints about the application of academic sanctions to students or others who object to such animal use. The potential for reputational damage resulting from such treatment of students was aptly illustrated by Rutgers University law professors Francione and Charlton in 1992, in their legal guidebook for such students and their lawyers, who commented in relation to such cases that, “*The conclusion that most people draw is an important and correct one: those who exploit nonhuman animals are often not reluctant to violate the civil rights of humans.*” [9] (p. x).

Such cases raise several ethical, legal and administrative issues of importance to veterinary and other biomedical faculties in which animals are used, including but not limited to those relating to animal welfare standards, applicable animal welfare and civil rights legislation, curricular design and professional accreditation requirements, the efficacy of both traditional and alternative teaching methods, academic freedom, and the non-discriminatory treatment of students.

Accordingly, within the last decade or more, some veterinary schools and universities have reviewed these issues in some detail, following which they have implemented policies allowing and formalizing the process of student conscientious objection to animal use considered harmful or objectionable. These have been called ‘conscientious objection’ or ‘student choice’ policies, with the latter being more common in North America. A recent publication, for example, described the establishment of such a policy at the recently established University of Adelaide’s School of Animal and Veterinary Sciences [10].

However, despite the introduction of such policies at several veterinary schools and universities within Australia, the US and elsewhere, very little has been published about the jurisprudential bases for student conscientious objection. Recommendations for the development of policy within this arena are similarly lacking. Instead veterinary schools have developed policy on an *ad hoc* basis, usually in response to a case or crisis in this area.

Accordingly, this paper provides pertinent information about the jurisprudential bases for student conscientious objection within Australia and the US, with some additional insights from international jurisdictions. It then describes the evolution of conscientious objection policies within Australian veterinary schools, and concludes with recommendations for the development and implementation of policy within this arena.

## 2. Definitions and Jurisprudential Bases for Conscientious Objection

### 2.1. Importance of Conscientious Objection within Democracies

Conscientious objection is generally considered to occupy a legitimate, and often legally protected, position within democratic societies. Within Australia for example, statutes making provision for conscientious objection include those pertaining to the vaccination of children, the participation of nurses in medical procedures, and attendance of voters at polling booths on days proscribed by their religion. Another example is provided by the Western Australian Equal Opportunity Act (1984) [11], which in some circumstances outlaws discrimination in education on the grounds of belief. The NSW Law Reform Commission Community Law Reform Program 6th Report *Conscientious Objection to Jury Service* [12], which examined this issue in detail, affirmed that,

*“Australian governments have often stressed the importance in a democratic system of respect for individual conscience”* (p. 27), and, *“It is fundamental to society that people should not be compelled to act against their consciences in the performance of civic duties except in matters of overriding importance or urgency such as a national emergency”* (p. 44).

This principle is also supported by Article 18 of the Universal Declaration of Human Rights, proclaimed by the General Assembly of the United Nations in 1948 [13], which asserts that,

*“Everyone has the right to freedom of thought, conscience, and religion; this right includes freedom to change his religion or belief, and freedom, either alone or in community with others and in public or private, to manifest his religion or belief in teaching, practice, worship and observance.”*

Additional international human rights legislation and the laws of several countries, e.g., England, India, Italy, The Netherlands and the US, support the rights of students to conscientiously object to participating in activities that run counter to their beliefs [9,14]. In 1993, for example, the Italian Parliament enacted a law recognizing the right of conscientious objectors to refuse to participate in animal experimentation and dissection. Similarly, Dutch universities are required to provide alternatives to students who do not wish to participate in exercises that harm animals. Likewise, a growing list of US states have passed laws requiring public schools to notify students (and/or their parents) that they may use alternatives to dissection or invasive live animal exercises without penalty [14].

### 2.2. Claims of Financial or Administrative Burden

These examples illustrate the fundamental importance normally accorded by modern democratic societies to the right of an individual to act in accordance with their conscience or belief. The “*national emergency*” case mentioned above provides a rare example in which violating such a right is considered acceptable. With respect to a conscientiously held student belief against participating in certain forms of educational animal use, as Francione and Charlton describe in their legal text on this issue [9], purported financial or administrative difficulties are not normally considered a sufficient reason for an educational institution to deny this student right, and to instead require that the student participate in such animal use on penalty of academic sanction. An institution may legitimately embark on such a course only if it can demonstrate that the financial and administrative burdens incurred by complying with such a student request would be so great as to seriously jeopardise the continued operation of the university or school—which could be contrary to the national interest. 

However, although a body of relevant case law exists from the US and several other countries [9] this author is unaware of any cases in which such an argument has been successfully raised in a legal setting. This is hardly surprising, because humane teaching methods are often cheaper than methods that rely on harmful animal use. Indeed, economic factors have been one of the most important forces driving the introduction of humane teaching methods internationally. Educational animal use is rarely cheap, after all: the purchase, transportation, housing, feeding, veterinary care when necessary, experimental anaesthesia, euthanasia and disposal of these animals, year after year, can add up to a considerable sum. Many alternatives, on the other hand, can be used largely cost free, for years, once the initial purchase has been made. Often the initial sum required is not prohibitive. Most computer simulations, for example, are available for a few hundred dollars or less, and surgical or clinical task trainers can be considerably cheaper. The financial advantages of alternatives have been demonstrated in several studies (e.g., [15,16]), and are likely to become increasingly important as economic pressures on universities continue to rise in many countries.

Similar benefits have been demonstrated in terms of time savings. In his description of nerve physiology experiments, Clarke [17] provides some insights as to why:

*“Previously, in such experiments, out of a typical allocated time of three hours, considerable time would be taken dissecting a viable sciatic nerve preparation and further time spent in trying to gain some small competence with the apparatus, at which point there would be a distinct possibility that the nerve was no longer viable (during the process of experimentation with the apparatus students often succeed in applying stimuli of enormous magnitude and frequency to the tissue). It is often a tired and irritable student who finally comes to the point in the experiment of measuring changes in response. Such a student is not in the optimum frame of mind to either perform the experiment with the due care and attention required or to think about the neurophysiological concepts involved. With the simulation, such problems are eliminated. Not only is much more time devoted to the experiment, but time is available to explore the subject in greater depth.”*

I can personally attest to the accuracy of the above comments, as this exact experiment was part of my veterinary physiology course when I was a student at Western Australia’s Murdoch University in 1998. The experiences of my classmates were very similar to those described. Dissection of the nerves beforehand by technicians lessened, but did not eliminate, the considerable problems involved.

To further support this point, studies such as that by Dewhurst and Jenkinson [18] have demonstrated that computer simulations save teaching time, are less expensive, and can be effective and enjoyable mode of undergraduate biomedical student learning.

Overall, existing studies demonstrate time and cost benefits, rather than disadvantages, associated with humane alternatives (additional examples include [19,20]). Hence, complying with reasonable student requests for humane alternatives would be highly unlikely to incur financial or other burdens sufficient to jeopardise the continued operation of the institution—the necessary criterion for legally denying such a request on the basis of such burdens.

### 2.3. Claims of Educational Necessity

An educational institution such as a veterinary school might also attempt to deny a student’s right to conscientiously object to certain animal use on the basis that such animal use is essential for educational purposes, and that without such use, the school would be unable to ensure the competency of the graduates produced. This is indeed valid with respect to some kinds of animal use, but not so with others. It is essential, for example, that veterinary students become competent at performing safe restraint, clinical examination and treatment of live animal patients. It is also essential that students gain sufficient knowledge of preclinical disciplines such as physiology, biochemistry and anatomy, as well as the practical skills required to provide surgical and medical interventions for patients. However, it does not necessarily follow that *harmful* animal use is required for the latter purposes.

A sizeable body of educational studies have compared the learning outcomes generated by non-harmful teaching methods with those achieved by harmful animal use. Of eleven studies of veterinary students published from 1989 to 2006 located during a recent survey [6], nine assessed surgical training. 45.5% (5/11) demonstrated superior learning outcomes using more humane alternatives. Another 45.5% (5/11) demonstrated equivalent learning outcomes, and 9.1% (1/11) demonstrated inferior learning outcomes. Twenty one studies of non-veterinary students in related academic disciplines were also published from 1968 to 2004. 38.1% (8/21) demonstrated superior, 52.4% (11/21) demonstrated equivalent, and 9.5% (2/21) demonstrated inferior learning outcomes using humane alternatives. 

Twenty nine papers in which comparison with harmful animal use did not occur illustrated additional benefits of humane teaching methods in veterinary education, including: time and cost savings, enhanced potential for customisation and repeatability of the learning exercise, increased student confidence and satisfaction, increased compliance with animal use legislation, elimination of objections to the use of purpose-killed animals, and integration of clinical perspectives and ethics early in the curriculum [6]. 

These studies clearly indicate that if humane alternatives are well designed, they normally achieve learning outcomes as good, or in well over a third of all cases, better than those that rely on harmful animal use.

Additionally, there are numerous veterinary and other biomedical courses worldwide where alternatives are extensively and successfully used. Finally, explicit forms of animal use are not normally required by accreditation authorities. For example, the Australasian Veterinary Boards Council—which accredits veterinary schools in Australia and New Zealand—merely provides more general guidelines, such as “*Institutions need to demonstrate that students have supervised, intramural exposure to the major production and companion animal species relevant to veterinary professional activities in Australasia*.” [21]. As long as the educational institution produces graduates demonstrably competent in a set of specified skills and other competencies, institutions usually have considerable freedom about how they choose to teach them.

For these several reasons, any institution that attempted to deny a student’s right to conscientiously object to harmful animal use on the basis of educational necessity would usually find itself in a weak position.

### 2.4. Adoption of Conscientious Objection Policies

Accordingly, veterinary schools and universities as a whole are increasingly acknowledging the validity of requests by students, and less often, faculty members, for humane teaching methods, and in several cases have established committees to examine these issues in depth. Policies allowing student conscientious objection, and formalising the process, have since been established at several veterinary schools. 

The first formal, written policy adopted in an Australian veterinary school appears to have been that adopted by Western Australia’s Murdoch University in 1998 (which has since been progressively updated; see Appendix), following a student campaign for humane teaching methods. The scope of this policy was not restricted to animal use, but covered any teaching or assessment activities to which students might conscientiously object, and was applicable to the university at large. Since then similar policies have been adopted at several other veterinary schools within Australia and abroad, such as those at the Universities of Sydney [22], Illinois at Urbana-Champaign [23], Queensland [24], and Adelaide [10], as well as by several other universities lacking veterinary faculties. In a majority of these examples conscientious objection policies are not restricted to the veterinary school, but are also applicable to other schools or programs, or to the university at large.

### 2.5. Definitions of Conscientious Objection

Should an institution choose to formulate a policy allowing student conscientious to certain forms of animal use, the definition of a conscientiously held belief will be one of its first, and most important, determinants. A definition that is too narrow in scope may prove insufficient to address legitimate student requests, prompting students to resort to the courts. One too broad may inadvertently allow a wider range of requests for alternate teaching and assessment activities than intended.

Fortunately, a body of legal precedent exists to inform such definitions. Australian courts have defined a conscientious belief as one based on a seriously and deeply held moral conviction, whether or not part of a religious doctrine or creed, and have stressed its durable, though not unchangeable, quality. The cases which have considered this issue have dealt primarily with conscientious objection to military service during the Vietnam War. Later cases adopting this definition have dealt with membership of Unions, and in a limited number of cases, with conscientious objection to jury service [25]. 

In two cases concerning conscientious objection to compulsory military service during the Vietnam War, the High Court of Australia approved a definition of conscientious objection previously provided by the Chief Justice of Western Australia [12]:

*“Conscientious belief is an individual's inward conviction of what is morally right or morally wrong, and it is a conviction that is genuinely held after some process of thinking about the subject. It represents a conclusion that is uninfluenced by any consideration of personal advantage or disadvantage either to oneself or others, and perhaps when put to the test should be ordinarily combined with a willingness to act according to the particular conviction reached although this may involve personal discomfort or suffering or material loss.”*

### 2.6. ‘Irrational’ Beliefs

Those unfamiliar with this field often assume that a conscientiously held belief must be necessarily religious or rational. However, conscientious beliefs pertain to the moral convictions of individuals. Perhaps because the reasons and reasoning underpinning moral viewpoints varies so much between individuals, applicable case law has not generally required such beliefs to be rational in the eyes of other people.

For example, the NSW Law Reform Commission [12] has asserted that:

*“In Australia various courts, tribunals and administrative officials have, over a substantial period, been required to test conscientious beliefs in the context of applications for exemption from civic duties imposed by legislation. The courts, in explaining this testing process, have been consistent in stating that the sole task of any person required to test a conscientious belief is to determine the genuineness of the particular applicant’s conviction and not to consider the reasonableness, wisdom or correctness of its content.”*

Similarly, the Chief Justice of Tasmania, in an early compulsory national service case [12], stated:

*“The only question I have to determine is whether the appellant does in fact conscientiously object to service … And if I find that he does then my own view of the cogency or otherwise of the reasons upon which he holds the objection becomes immaterial, since it is of the essence of freedom of conscience that a man may hold to his conscientious conviction irrespective of whether a Judge or any other person thinks that he ought. Nor do I think that I should be too ready to impugn the bona fides of his objection because of some inconsistency in the views which he puts forward, or of evidence of instances of divergence between his behavior and his principles, since the compatibility of such phenomena with sincerity is unfortunately a commonplace of human experience.”*

This statement has been expressly approved by the Supreme Court of South Australia. 

### 2.7. The Role of Religion

Similarly, although conscientiously held beliefs may indeed be religious, they are not required to be so to qualify as conscientiously held. This principle is not immediately obvious when examining some applicable legislation. Anti-discrimination (or ‘equal opportunity’) legislation, for example, often outlaws discrimination in the workplace or educational settings on the basis of political and religious beliefs. Discrimination on the basis of other beliefs is often not explicitly prohibited. The Western Australian Equal Opportunity Act (1984) [11] provides one such example. 

However, as stated by the NSW Law Reform Commission Report [12],

*“Some legislation has addressed religious objections exclusively. This reflects a view that a distinction can properly be drawn between a conscientious belief based on a religious doctrine and a conscientious belief not so based. However, the decided cases have affirmed that the term “conscience” of itself is not to be restricted by the ambit of “religion”. Nor is the term “religion” to be defined restrictively. The Chief justice of the High Court of Australia warned in the Jehovah’s Witness Case that “each person chooses the content of his own religion” and “[i]t is not for a court, upon some a priori basis, to disqualify certain beliefs as being incapable of being religious in character.”* (pp. 27–28).

This principle has been upheld by US courts, which wisely chose not to enter into the social debate about which beliefs qualify as religious—a debate the religious community itself has been unable to fully settle, despite hundreds of years of effort.

The NSW Law Reform Commission Report further states on p. 45 that,

*“There appears to be no persuasive reason to restrict exemption to objectors on religious grounds. We consider that to do so would unjustifiably discriminate against those with sincere moral convictions which are unrelated to religious tenets.”*

This principle has been upheld in most Australian states when considering conscientious objection to jury service. Australia's Defence Legislation Amendment Act (1992) [26] states in Section 4 with respect to *“exemption from service because of conscientious beliefs… from combatant duties”*, that:

*“a person is taken to have a conscientious belief in relation to a matter if the person’s belief in respect of that matter:*

(*a*) *involves a fundamental conviction of what is morally right and morally wrong, whether or not based on religious considerations; and*
(*b*) *is so compelling in character for that person that he or she is duty bound to espouse it; and*
(*c*) *is likely to be of a long standing nature.”*

### 2.8. Definition of Conscientious Objection

Based on the jurisprudential principles previously described, a conscientiously held belief against participating in any nonessential teaching or assessment activity should comply with the following elements:

- Such a belief should represent be an individual’s inward conviction of what is morally or religiously right or wrong.
- Such a belief need not qualify as rational in the eyes of others, or as a religious belief, although both may be true.
- Although when put to the test a conscientiously held belief should ordinarily be combined with a willingness to incur personal discomfort or suffering or material loss, the essence of non-discriminatory principles is that no student should be required to incur such losses, as a result of conscientiously objecting to participation in any nonessential educational activity. For the reasons provided previously, this clearly includes teaching or assessment activities involving harmful animal use.

## 3. Managing Conscientious Objection

Once agreement has been reached about which beliefs may be defined as conscientiously held, universities must decide how to assess conscientious objection claims, and what to do when a student request for alternate teaching and assessment activities is found to be based on conscientiously held belief. A range of approaches to these issues have been adopted by veterinary schools and universities.

### 3.1. Restrictive Approaches

A very few universities have chosen to adopt policies attempting to restrict enrolment to students that do not have conscientious objections to participating in harmful animal use within curricula. Examples include:

- Requiring students to sign an agreement on enrolment to the effect that they will participate in potentially objectionable activities, such as the dissection of purpose-killed animals, or experimentation on living animals.
- Requiring students to register their conscientious objection at time of application or enrolment.
- Screening students via their application forms and interviews in an attempt to exclude students who could conscientiously object to harmful animal use.

Adoption of such approaches potentially incurs several problems, however. First, if explicitly stated, such clearly discriminatory policies leave universities vulnerable to legal challenges. For example, as stated previously, in some circumstances the Western Australian Equal Opportunity Act (1984) [11] outlaws discrimination in education on the grounds of belief. 

The publication of requirements that students must dissect purpose-killed animals, or experiment on living animals, also brings considerable potential for adverse publicity. The latter point is one universities are generally sensitive to, and was clearly illustrated in 2008 at Western Australia’s Murdoch University. Within a short space of time Murdoch’s attempt to require participation by two students in such harmful animal use resulted in significant adverse coverage for the university in both national television and national print media. Similar cases have occurred elsewhere.

Furthermore, students applying for admission to highly competitive courses such as veterinary science can be expected to be reluctant to admit any such concerns at the time of application or enrolment, reasonably fearing prejudicial treatment. An accurate picture could not be gained in this way.

Finally, one of the objectives of a university education is to teach students to think critically. It is a particular goal of most veterinary curricula to educate students about animal welfare issues, and to encourage students to think critically about these. Accordingly, it is hardly surprising that the beliefs of some students may evolve, and indeed change, over the duration of their veterinary courses, as they gain relevant knowledge and experience, and as their critical thinking skills progress. 

Students initially supportive of harmful animal use within curricula may sometimes find themselves increasingly sceptical, and then opposed to such use. Such was the case at New Zealand’s Massey University veterinary school when the opinions of third, fourth and fifth year veterinary students were surveyed in 2001, regarding the learning value of terminal physiology labs conducted in the third year of their veterinary curriculum [27]. In every one of nine questions about the educational value and ethical justification for these laboratory exercises, the attitudes of the fifth year students differed significantly from those of third year students. The authors concluded that, “*as the vet students are gaining experience their opinions appear to move systematically away from the position they held in year three.”* And, *“…the experiences of the vet students appear to be inducing doubt as to how justifiable is the use of live animals, relative to the knowledge and skill gained from the practise.*” Prior to this survey 68 sheep were killed annually. Partly due to these results, Massey University stated its intention to phase out these laboratories by 2004.

### 3.2. Inclusive Approaches

Other universities have formalized explicit policies welcoming students with a more diverse range of beliefs. Sometimes this is included within more general policies affirming equal educational opportunities for students from a diverse range of religious, cultural or other backgrounds. The discussion paper on student conscientious objection circulated at Murdoch University in 1998 provides a good example:

“*The university is a public body whose mission includes a commitment to equity and to caring for its students”,* and, *“By our very nature as a university, we are also committed to welcoming and respecting a range of ideas and beliefs, including those with which we may disagree.*”.

[25]

This ethos is reinforced by further statements within Murdoch’s Handbook such as, “*Murdoch University is committed to equal opportunity and social justice principles, policy and practice through embedding social justice into academic business, maintaining an environment free from discrimination and supporting the diverse needs of students and staff.*” [28]. Murdoch has a dedicated website (http://our.murdoch.edu.au/Equal-opportunity-and-social-justice/) and a Student Equity and Social Justice Committee dedicated to realizing such ideals.

Such inclusive approaches have obvious benefits. They foster multiculturalism, increased understanding of, and tolerance for, differences, and may also yield public relations benefits in these respects. They are significantly more likely to comply with anti-discrimination legislation. They are more encouraging of the honest expression of student viewpoints on animal use issues, which in turn facilitates more honest discussion of these issues within animal welfare courses and elsewhere, creating better opportunities for student learning about animal welfare and animal ethics. And they avoid the potential for negative outcomes, including court challenges and adverse publicity, associated with more restrictive approaches.

### 3.3. Policy Recommendations

The *ad hoc* approach of many universities to conscientious objection frequently results in student requests for humane alternatives being made at late notice, increasing the likelihood such requests will be denied, and the chances of subsequent conflict; or alternatively, increases the likelihood that any alternatives provided will be suboptimal, due to insufficient preparation time. A formal policy and process pertaining to conscientious objection is therefore recommended, as it avoids such ‘crisis management’, and allows superior preparation and planning.

Social views on the acceptability of invasive curricular animal use are evolving, and it is predictable that student requests for humane alternatives are likely to increase, where such animal use remains. Accordingly, it is recommended that universities which include such curricular animal use should adopt and implement a formal policy allowing conscientious objection to educational activities that violate the conscientiously held beliefs of students. Alternative educational experiences and assessments should be provided for these students with the aim of providing equivalent outcomes in terms of knowledge and/or ability. These should require approximately equal commitments of time and effort, and in particular, should not be punitively burdensome, with the capacity to demonstrate this in case of scrutiny or challenge. 

Although universities should generally accommodate requests for alternate teaching or assessment activities where participation would require a student to violate a conscientiously held belief (such as against unnecessarily harming animals), this does not mean that all requests should be accommodated. In particular, universities should not accommodate requests if by doing so they would violate a law (e.g., demands for racial segregation due to beliefs based on racism).

Finally, in the event that a student’s request is denied or the student is unsatisfied with the alternative offered, an appeals process should be available.

### 3.4. Publication of Policies

Well prior to the commencement of any course in which animals are used, information about animal use and the university policies pertaining to conscientious objection should be published in writing to all students taking applicable courses. This information should be provided within student handbooks, curricular and course guides, and any other appropriate information outlets.

The university handbook should include a section on conscientious objection, which details the conscientious objection policy, provides the university definition of a conscientiously held belief, describes the procedure for registering a conscientious objection to participating in a teaching or assessment activity, and the procedure for assessing claims of conscientious objection, and details the appeals process. 

Students should be advised to seek information as early as possible about educational activities to which they might have a conscientious objection, from any applicable curricular guides, and particularly, any course descriptions or materials such as laboratory descriptions, as well as from the student handbook, Course Coordinators, or their Program Chairs or Heads of School. Students should be advised of the benefits of early notification; particularly, that this will allow sufficient time for their claims to be assessed, and for alternatives to be sourced and prepared, and will decrease the likelihood that suboptimal activities will be provided. Students should be requested to raise their claims of conscientious objection with their Course Coordinators, Program Chairs or Heads of School as early as possible, and preferably before the start of semester, although this will be determined to some extent by the date of release of course materials describing curricular animal use and conscientious objection policies.

To facilitate this process, course and curricular materials should aim to summarise information on potentially objectionable activities occurring within any courses. In the case of animal usage, this information should include all of the following:

- which courses animals are used in
- what species and numbers of animals are used
- how the animals are sourced
- a summary of the procedures carried out on them (e.g., dissection, experimentation on living animals or organs sourced from them, non-recovery surgery)
- why the animals are considered necessary
- details of any refinement methods used (to decrease suffering, and maximise well-being, such as analgesics or environmental enrichment)
- details of their housing or caging on or off campus, prior to and after use
- whether and how the animals are euthanized and disposed of

All of this information may be necessary to allow students to properly consider whether or not they conscientiously object to activities involving these animals. 

A minority of poorly prepared students may only become aware of the depth of their objection to an activity when physically confronted with it (e.g., in the case of highly invasive experiments on living animals), and the university may still be obliged to cater for them. Therefore, there the absolute cut-off date for raising claims of conscientious objection should be the date of the objectionable activity in question. It is not reasonable for any student to request an alternative to an activity in which they’ve already chosen to participate, on the basis that they conscientiously objected to it. It is important to bear in mind that this defence may not apply, however, if a student is able to reasonably claim they were coerced into participating, by the application of any undue pressure by faculty, for example. However, it should be made quite clear that the quality of alternatives provided to students may well depend on the amount of time they give staff to prepare them. Staff should also be directed to consider in advance what alternatives they might provide, should students raise objections to participating in activities within their courses.

### 3.5. Assessing Claims of Conscientious Objection

Claims of conscientious objection should be initially assessed by the Course Coordinator, or, where the issue is systemic to the units offered within a programme, by an appropriate official such as the Programme Chair or Head of School. 

Students voicing a conscientious objection should normally be initially required to meet with the appropriate official concerned, who should seek to clarify what activity the student is seeking to object to, exactly which aspects of the procedure they are objecting to (e.g., the species used? the invasiveness of the procedure? the killing of an animal?), and whether any mitigating factors might be sufficient to overcome these objections, such as the use of a species of lower sentience, a less invasive procedure, or the use of analgesics or anesthetics. The latter information might be important in seeking to establish an acceptable alternative teaching or assessment activity. To determine whether the objection(s) are truly conscientiously held, or merely elicit feelings of discomfort, for example, the official should also seek to determine the depth of the student’s convictions, by careful questioning. As stated previously, an acceptable definition of a conscientiously held belief is that when put to the test such a belief should ordinarily be combined with a willingness to incur personal discomfort, suffering or material loss. However, the essence of non-discriminatory principles is that no student should be required to incur such losses, as a result of their conscientiously objection.

To increase the transparency and defensibility of these proceedings, it is also advisable to ensure at least one additional faculty member or university official is present as a witness. The student should also be offered the opportunity to invite a friend, colleague or family member in support. 

At the beginning of the meeting, the process for assessing claims of conscientious objection, and in particular the type of questions to be asked and the need to ask them, should be explained to all parties, who should also be reminded of the university’s commitment to maintaining the confidentiality of personal information pertaining to students. In the interest of maintaining written case records, at the end of the meeting students should perhaps be asked to follow up with a written description of their conscientious objection and a formal request for alternatives. At the end of this process of assessment a decision should be made about what alternatives will be offered to accommodate the student’s request, or to instead deny the request.

Students whose claims are denied or who are dissatisfied with these assessments and educational experiences subsequently offered should have access to an avenue of appeal. Given the potential legal implications for the university where conscientious objectors are discriminated against, appeals committees should include a lawyer, ideally with relevant expertise, possibly from the university’s law faculty, if such exists.

### 3.6. Related Considerations

Faculty members assessing claims of conscientious objection should be appraised of the details of the university’s conscientious objection policy, including the agreed definition of conscientiously held beliefs, the procedure for assessing claims of conscientious objection, and of the appeals process. In particular such faculty should be reminded that conscientious beliefs need not have a religious or rational basis, and of the desirability of showing respect to all beliefs, including those with which they might personally disagree. Their sole objectives should be to determine whether or not the beliefs in question are conscientiously held, and if so, what alternatives to the objectionable activities can be provided, given concurrent requirements to ensure the curriculum continues to meet any applicable accreditation standards. Faculty should understand that they must not seek to cross examine students unduly, nor seek to alter their beliefs.

Finally, records should be kept of all cases of student conscientious objection, sufficient to support a defence in the event of legal challenge, as well as to support educational studies, such as those examining student learning outcomes using different teaching methodologies. These should include, at a minimum, details such as the nature of the conscientiously held belief(s), the activities objected to, alternatives provided, educational performance of the student at that time, at the end of the course, and within the degree overall as assessed at the time of graduation, as well as the dates of all relevant events, and the names of all involved.

## 4. Conclusions

The right of an individual to act in accordance with their conscience or belief is often considered fundamentally important within modern democracies, with the result that it may legally be violated only in the gravest of circumstances, such as national emergencies. With respect to student (or, less commonly, faculty) beliefs against participating in activities involving animals, it is likely that a university could legally require such conscientious objectors to violate their beliefs on penalty of academic sanction, only if it could demonstrate to the satisfaction of a court that participation in such activities was truly necessary to ensure the competency of the graduates produced, or alternatively, that accommodating such beliefs would incur financial or administrative burdens so severe as so seriously threaten the continued operation of the institution. However, when alternatives are requested to harmful animal use, a considerable body of existing evidence suggests financial and time-related benefits, rather than disadvantages, associated with humane teaching methods. Because of such factors, it highly unlikely such a case could be won in a court of law. 

Additionally, because of changing knowledge pertaining to the sentience and other psychosocial characteristics relevant to the moral status of animals, as well as changing social attitudes toward animals and changing student demographics, and the on-going development of humane teaching methods, conscientious objection to harmful animal use within veterinary and other biomedical faculty are likely to continue to increase over time.

Accordingly, it is recommended that those universities offering relevant courses that have not yet done so, proceed to implement policies agreeing to make reasonable accommodations for students (or staff) who conscientiously object to participating in harmful animal use.

Formalisation of such policies has several advantages. Such formal policies demonstrate institutional commitment to fostering a culture which is tolerant of diversity, and respects a range of viewpoints, beliefs and backgrounds. They can increase compliance with applicable legislation outlawing certain forms of discrimination in education or the workplace. They greatly decrease the likelihood of conflicts relating to curricular animal use, which can be extremely damaging to the careers of the students or others involved, and to the reputation of the university at large. And they maximise the likelihood of honest disclosure of student concerns, and of prior warning of incidents, and minimise crisis management or *ad hoc* responses.

Once adopted, detailed information about curricular animal use and related conscientious objection policies, including the appeals process, should be publicised to all students via university handbooks, curricular and course guides, and any other appropriate information outlets, well prior to the commencement of courses in which animals are used. Such information should also be circulated to faculty, along with guidelines about assessing conscientious objection claims, and the provision of alternative teaching or assessment activities. Faculty should be reminded that applicable case law does not require conscientiously held beliefs to be rational or religious in the eyes of other people, and of the wisdom and necessity of not exerting undue pressure on such students, and of showing respect for beliefs with which they may personally disagree. 

## Appendix

**Conscientious Objection in Teaching and Assessment Policy**

**Murdoch University, Western Australia**

Initially approved 11 November 1998

Current version approved 8 August 2012 [29]

### Purpose

To provide guidance to students and staff dealing with situations where conscientious belief conflicts with unit requirements.

### Policy

1. The University recognises that some students may have a conscientious belief which is in conflict with learning activities, including those for assessment, in one or more units in which they enrol. The University shall endeavour to make reasonable accommodations to meet such beliefs. Notwithstanding the provisions of this policy, the University will not act in any way that violates Commonwealth or State law and the University is not obliged to accommodate a conscientious belief which puts it at risk of violating a law (e.g., a belief based on racism).
2. In considering such cases, the University accepts that conscientious belief is a genuine and sustained conviction of what may be morally right or wrong that is uninfluenced by any consideration of personal advantage or disadvantage to either the student themselves or others in pursuit of their course of study. This conviction can be based on religious reasons, belief in the sanctity of life, environmental concerns, or other reasons that the student deems central to their belief system.
3. The onus is on the student to take the initiative in identifying a conscientious difficulty with a learning activity, including those for assessment and to draw this to the attention of the University before undertaking such practice. [A student cannot appeal against a practice which he or she has already undertaken.] It is preferable for students with a conscientious objection to be identified early, so there is time to assess it and to make any necessary arrangements. Wherever possible, students with a conscientious objection in a unit should raise their difficulties with the Unit Coordinator prior to the start of the unit or in the first three weeks of semester. If the difficulty is with units in future semesters or is systemic to units offered in the course, the student should discuss this with the Academic Chair as early as possible. It is for these staff to assess whether the claim constitutes a conscientious objection and what arrangementscan be made to accommodate it. The staff member has the discretion to ask for more information from the student in order to establish whether or not the student has a conscientious belief.
4. In cases where Unit Coordinators can foresee students having problems of belief in their unit, the unit study guide should mention these and advise any students with problems about this to see the Unit Coordinator.
5. The student can request that there be a suitable alternative, but has no right to demand that the alternative take a particular form. There are also countervailing factors to be taken into account in deciding whether and (if so) how to meet the student’s concerns, including:
  - professional requirements: those of external registration bodies, and staff concerns to be able to certify that graduates have met the course learning outcomes and basic professional competencies. This requires a careful consideration of whether or not the learning activity or assessment at issue is essential for the training of practitioners in that profession.
  - whether it is a required or an elective unit (the case for expensive alternative arrangements in an elective unit is much weaker)
  - whether there is time to put alternative arrangements in place
  - whether it would result in the University breaching its equal opportunity obligations
  - whether other students would be disadvantaged in the quality of their education
  - cost.
6. Students with a conscientious objection to a particular learning activity, including those for assessment, should not simply be excused from an activity, but instead be given an alternative that meets the same learning outcomes. Alternatives made available to students with a conscientious objection do not have to be made available to all other students in the unit.
7. A Unit Coordinator who has considered and approved a student case of conscientious objection must advise the Enrolments and Fees of this, giving details of the nature of the conscientious belief and the alternative arrangements made for loading into the student record system.
8. Unit Coordinators should ensure that the alternative arrangements made for similar conscientious objections are consistent.
9. A student who is dissatisfied with the decision of the Unit Coordinator may request the School Dean to review the decision and thereafter appeal to the Student Appeals Committee.
